# The potential effectiveness of the nutrition improvement program on infant and young child feeding and nutritional status in the Northwest and Southwest regions of Cameroon, Central Africa

**DOI:** 10.1186/s12913-016-1899-z

**Published:** 2016-11-15

**Authors:** Kate Reinsma, Godlove Nkuoh, Emmanuel Nshom

**Affiliations:** 1 Nutrition Improvement Program, Cameroon Baptist Convention Health Services, P.O Box 1, Nkwen, Bamenda, Cameroon; 2 AIDS Care and Prevention Program, Cameroon Baptist Convention Health Services, Bamenda, Cameroon

## Abstract

**Background:**

Despite the recent international focus on maternal and child nutrition, little attention is paid to nutrition capacity development. Although infant feeding counselling by health workers increases caregivers’ knowledge, and improves breastfeeding, complementary feeding, and children’s linear growth, most of the counselling in sub-Saharan Africa is primarily conducted by nurses or volunteers, and little is done to develop capacity for nutrition at the professional, organizational, or systemic levels. The Cameroon Baptist Convention Health Services Nutrition Improvement Program (NIP) has integrated a cadre of nutrition counselors into prevention of mother-to-child transmission of HIV programs, infant welfare clinics, and antenatal clinics to improve infant and young child feeding practices (IYCF). The study objective was to evaluate the effects of NIP’s infant feeding counselors on exclusive breastfeeding (EBF), complementary feeding (CF), and children’s linear growth.

**Methods:**

A cross-sectional evaluation design was used. Using systematic random sampling, caregivers were recruited from NIP sites (*n* = 359) and non-NIP sites (*n* = 415) from Infant Welfare Clinics (IWCs) in the Northwest (NWR) and Southwest Regions (SWR) of Cameroon between October 2014 and April 2015. Differences in EBF and CF practices and children’s linear growth between NIP and non-NIP sites were determined using chi-square and multiple logistic regression.

**Results:**

After adjusting for differences in religion, occupation, and number of months planning to breastfeed, children were almost seven times (Odds Ratio [OR]: 6.9; 95% Confidence Interval [CI]: 2.30, 21.09; β = 1.94) more likely to be exclusively breastfed at NIP sites compared to non-NIP sites. After adjusting for differences in occupation, religion, number of months planning to breastfeed, rural environment, economic status, attending other Infant Welfare Clinics, and non-biological caregiver, children were five times (OR: 5.5; CI: 3.37, 9.02; β = 1.71) more likely to be stunted at non-NIP sites compared to non-NIP sites.

**Conclusion:**

Training a cadre of nutrition counselors is one approach towards increasing nutrition human resources to implement nutrition interventions to improve maternal and child nutrition. In this research project, the study design did not allow for conclusive results, but rather suggest IYCF counseling provided by nutrition counselors was effective in increasing EBF and reduced the risk of stunting in children 6–8 months.

**Electronic supplementary material:**

The online version of this article (doi:10.1186/s12913-016-1899-z) contains supplementary material, which is available to authorized users.

## Background

International consensus is building to address maternal and child undernutrition since sluggish improvement is an underlying factor in the diminished achievement of the Millennium Development Goals 4, Reduce Child Mortality and 5, Improve Maternal Health [1]. In 2010 the Scaling Up Nutrition (SUN) network emerged to support national governments to align nutrition programs around common objectives and in 2012 the World Health Assembly endorsed a Comprehensive Implementation Plan on Maternal, Infant and Young Child Nutrition, focusing on reducing wasting, stunting, anemia, overweight, low-birth weight and improving exclusive breastfeeding. To help communities achieve the six global nutrition targets, the World Health Organization (WHO) outlined 5 priority actions, one of which designated increasing human resources to implement nutrition interventions [2, 3].

Although there is renewed international focus on nutrition, little attention is paid to human resource development [4], even though it is well-acknowledged that lack of human capacity in nutrition is one of the factors hindering African countries from achieving the global nutrition targets [5]. Although infant feeding counseling by health workers increases caregivers’ knowledge, breastfeeding and complementary feeding practices, as well as improve children’s linear growth and weight gain [6–10], most of the counseling in sub-Saharan Africa is primarily conducted by mid-level nurses or community volunteers, not by nutrition educators/counselors, [8, 9], and little is done to develop capacity for nutrition at the professional, organizational, or systemic levels [5, 11–13].

Cameroon is a low-to-middle income, sub-Saharan African country bordering Nigeria, Chad, Central African Republic, Gabon, and the Congo. In March of 2013 Cameroon joined the SUN movement and the government pledged to develop a multisectoral policy for nutrition, align nutrition programs toward a common results framework, and improve clarity over investments in nutrition and budgeting [14]. Despite nutrition policy improvements, malnutrition remains a serious public health problem in Cameroon. Approximately 15% of children 0–5 years of age are underweight, 33% are stunted, and 5.6% are wasted. Only 20% of infants between 0 and 6 months of age are exclusively breastfed and 69% received solid or semi-solid foods at 6–8 months [15]. Micronutrient deficiencies are common, 60.3% of children and 39.5% of pregnant women experience iron-deficiency anemia [16]. At the same time, urbanization, demographic, and economic changes have led to a nutrition transition, characterized by higher caloric content and/or reduction in physical activity, resulting in increased levels of obesity and hypertension in adults [17, 18].

Similar to other African countries, Cameroon is facing a crisis of inadequate recruitment, management, and distribution of health personnel, likely impeding Cameroon’s progress in reducing under- and overnutrition [19, 20]. Although stakeholders agree on the urgent need to develop capacity for nutrition, country-wide initiatives so far have been limited [20].

In an effort to ameliorate the shortage of human resources in nutrition, the Cameroon Baptist Convention Health Services (CBCHS) started a Nutrition Improvement Program (NIP). Since 2007, NIP has trained and integrated a cadre of nutrition counselors into prevention of mother-to-child transmission (PMTCT) of HIV programs, infant welfare clinics (IWC), and antenatal clinics (ANC) to improve infant and young child feeding (IYCF) practices. NIP is unique because it utilizes people who have passed Cameroon’s General Certificate Examination (GCE) in Food and Nutrition and trains them in evidence-based practices for nutrition counseling and assessment with ongoing supervision by nursing and nutrition supervisors. To our knowledge, no other health system in Africa has auxiliary nutrition personnel integrated into IWC, ANC, Maternities, and Outpatient clinics whose sole purpose is to specifically provide nutrition counseling on malnutrition, breastfeeding, complementary feeding, growth monitoring, management of breast difficulties, breastfeeding attachment, and chronic diseases such as hypertension, HIV, and type II diabetes. Nutrition counselors participate in a 3 month intensive course covering topics such as infant and young child feeding in the context of HIV; HIV testing and counseling; promoting of exclusive breastfeeding; management of breastfeeding difficulties; proper positioning and attachment to the breast; provision of F75/F100 for malnourished children; preparation and demonstration of supplementing pap (common infant cereal in Cameroon) with soy beans and/or peanut butter; and nutritional management of obesity, diabetes, hypertension, and gout. After the training, counselors are posted to hospitals for a 3-month internship. After the internship, if the counselors adequately demonstrate nutrition counseling skills, they are posted to a CBCHS institution. The purpose of this paper is to evaluate the potential effectiveness of the Nutrition Improvement Program on infant and young child feeding practices and nutritional status in the NW and SW Regions of Cameroon. The objectives of the evaluation were:

1. To determine the potential effectiveness of infant feeding counseling on exclusive breastfeeding rates in children between the age of 0–5 months
2. To determine the potential effectiveness of infant feeding counseling on complementary feeding in children between the age of 6–8 months
3. To determine the potential effectiveness of infant feeding counseling on wasting (weight-for-height z score) and/or stunting (height-for-age z score) in children 0–8 months

## Methods

### Study design

This was a comparative cross-sectional evaluation.

### Study population

The study population were caregivers, between the ages of 18–50, and their infants between the ages of 0 and 8 months. Caregivers were recruited from three existing Northwest Region CBCHS NIP sites (one urban hospital, one rural hospital, one rural health center) and one existing Southwest Region CBCHS NIP site (one urban hospital). Comparison group participants came from non-NIP sites (one urban hospital and one rural health center in the Northwest Region; one urban health center and one rural health center in the Southwest Region) and matched for demographics. Both regions, from which participants were recruited, are tropical in climate with year-round access to food and the majority of residents rely on subsistence farming and attain at least a primary-level education.

### Sampling and data collection

According to the WHO Global Databank on Infant and Young Child Feeding, the average percent of infants who were exclusively breastfed and complementary feeding practices in the NW and SW Regions was 34 and 78% respectively [21]. In the sample size calculations these statistics were used as a proxy measure of exclusive breastfeeding and complementary feeding practices at non-NIP sites. According to the NIP records for the month of January 2014, 78% of children were exclusively breastfed and 85% received complementary foods. Comparing the probabilities from NIP and non-NIP sites at a power of 90%, an alpha level of .05, 10% attrition, and an equal allocation ratio for a logistic regression test, a total sample size of 130 NIP and non-NIP caregivers with children between the ages of 0–5 months and 630 NIP and non-NIP caregivers with children between the ages of 6–8 months was needed. The statistical software program G*Power was used to determine sample size [22].

**Fig. 1:**
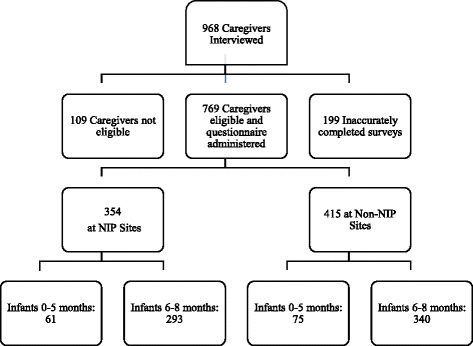
Data Collection

Caregivers who presented at the IWC clinics during the data collection periods were recruited through systematic random sampling using a sampling interval, which was calculated based on the average daily attendance at IWCs. Every person recruited was given the opportunity to participate or not participate in the evaluation and assured of the same quality of health care. At NIP sites there were 354 caregivers and at non-NIP there were 415 caregivers who completed the questionnaire October 2014-April 2015. Figure 1 demonstrates how data was collected from NIP and non-NIP sites for caregivers with children between 0–5 and 6–8 months.

An adapted validated IYCF questionnaire was used to determine exclusive practices breastfeeding, timing of introduction to complementary feeding [23], and demographic information (please see Additional file 1). The IYCF questions were modified to reflect common locally available foods (pap, fufu, njamajama) and collect relevant demographic data. The demographic variables such as caregiver’s religion (Muslim, Christian, or other), gender, years of education, occupation, number of children, marital status, region of residence (NW or SW), location of health center (rural or urban), number of months planning to breastfeed, attendance of IWC at other health centers, and economic status were gathered from the questionnaire. The ownership of a radio, television, motorbike, and car were summed to determine economic status. This method of measuring economic status was found effective in a Cameroonian study that sought to determine child nutritional status by household and community socioeconomic status [24]. Prior to data collection, the questionnaire was pre-tested on a small sample and further revised to suit the Cameroonian context. Data collectors were trained to verbally administer the questionnaires to eligible caregivers and collect their children’s anthropometric measurements at the Infant Welfare Clinics (IWCs) at four NIP sites and four non-NIP recruitment sites.

To determine children’s nutritional status, anthropometric measurements (weight-for-height, height-for-age) were collected using standardized procedures to permit reproducibility and accuracy. When infants came to the IWC, they were weighed naked on a baby scale measured to the nearest 10 g. Recumbent length was measured to the nearest millimeter using horizontal measuring boards. All infant weight and height measurements were taken in duplicate by the data collectors and if there were any differences between measurements greater than 0.1 kg in weight and .1 cm in height, a third measurement was taken to ensure accuracy. The age of the child was determined using the child’s health record when available. When not available, the child’s age was obtained from the caregiver. The infant’s anthropometric measurements were recorded on the questionnaire. The WHO 2006 indices for weight-for-height and height-for-age were used for comparison.

### Outcome measures

Exclusive breastfeeding under 6 months was defined as the proportion of infants 0–5 months of age who were fed exclusively with breastmilk the previous day. Complementary feeding was expressed as the introduction of solid, semi-solid or soft foods between 6 and 8 months. The 24-h recall method was used to measure the proportion of infants who were exclusively breastfed or received complementary foods in the past 24 h, per the World Health Organization’s standards [23].

If a child’s weight-for-height z-score was below 2 standard deviations, the child was considered wasted. If the child’s height-for-age z-score was below 2 standard deviations, the child was considered as stunted. The child’s anthropometric measurements were analyzed to determine if there was a difference in children’s nutritional status between those who did and did not receive NIP counseling.

### Statistical analysis

The WHO Anthro Software 3.2.2 was used to determine if a child was stunted or wasted based on their height for age and weight for height z-score. Chi-square was used to measure differences in demographics, exclusive breastfeeding, complementary feeding, stunting, and wasting between caregivers and their children who received services at NIP sites and those who received services at non-NIP sites. Binary logistic regression determined if caregivers at NIP sites were significantly more likely to exclusively breastfeed or provide timely complementary foods and if children at NIP sites were less likely to be stunted or wasted after adjusting for confounders. Caregivers with children 0–5 months were analyzed separately from caregivers with children 6–8 months. All data was analyzed using SPSS version 23 [25].

## Results

Of the 968 caregivers who were recruited, 769 completed the infant feeding surveys. Inaccurately completed surveys by data collectors (199) and surveys completed by caregivers who did not have a 0–8 month-old child or were under 18 years (109) (the age to provide legal consent to participate in research studies in Cameroon) were excluded from the analyses (Fig. 1).

### Sample characteristics

The demographic characteristics of caregivers who completed the survey are illustrated in Table 1. Compared to caregivers of children 0–5 months at non-NIP sites, there were significantly more Muslims, more health workers, and fewer Civil Servants among caregivers of children 0–5 months at the NIP sites. Compared to caregivers of children 6–8 months at non-NIP sites, there were significantly more who were Muslims, farmers, receiving services from a rural health centers, attending IWC at other health centers, and had lower economic status among caregivers of children 6–8 months at the NIP sites. Furthermore, caregivers of children 0–5 and 6–8 months at NIP sites also planned to breastfeed significantly longer than caregivers at non-NIP sites (*p* = 0.02, *p* = 0.00). The differences in demographics and breastfeeding intention and duration between caregivers from NIP and non-NIP sites were included as confounders and adjusted for in the binary logistic regression analysis.

**Table 1:** Participants with Selected Demographic Characteristics

Caregivers with Children 0-5 Months			
	NIP = 61 (*n*) %	Non-NIP = 75 (*n*) %	*p*
Marital Status			
Married/Cohabitating	(51) 85%	(57) 76%	0.19
Singe/Widowed	(9) 15%	(18) 24%	
Biological Mother or Father			
Yes	(57) 93.4%	(65) 86.7%	0.20
No	(4) 6.6%	(10) 13.3%	
Gender of Caregiver			
Male	(0) 0%	(1) 1.3%	0.36
Female	(61) 100%	(74) 98.7%	
Religion^a^			
Muslim	(10) 16.7%	(1) 1.3%	0.00
Christian	(50) 83.3%	(74) 98.7%	
Other			
Occupation^a^			
Self-employed	(18) 29.5%	(31) 41.3%	0.02
Civil Servant/Business	(6) 9.8%	(12) 16%	
Health worker	(10) 16.4%	(1) 1.3%	
Student	(7) 11.5%	(10) 13.3%	
Housewife/Farmer	(20) 32.8%	(21) 28%	
Location			
Urban	(27) 44.3%	(35) 46.7%	0.78
Rural	(34) 55.7%	(40) 53.3%	
Region			
Northwest	(41) 67.2%	(50) 66.7%	0.95
Southwest	(20) 32.8%	(25) 33.3%	
Child attended IWC elsewhere			
Yes	(27) 44.3%	(26) 34.7%	0.25
No	(34) 55.7%	(49) 65.3%	
Mean Age of Caregiver			
	27.2	27.33	0.89
Mean Sum of Economic Variable			
	1.75	1.87	0.57
Mean number of years of education			
	11.74	10.44	0.39
Number of months planning to breastfeed^a^			
	21.88	13.28	0.02
Caregivers with Children 6-8 Months			
	NIP = 293 (*n*) %	Non-NIP = 340 (*n*) %	*p*
Marital Status			
Married/Cohabitating	(234) 81%	(269) 79.4%	0.61
Singe/Widowed	(55) 19%	(70) 20.6%	
Biological Mother or Father^b^			
Yes	(263) 93.3%	(327) 97%	0.03
No	(19) 6.7%	(10) 3%	
Gender of Caregiver			
Male	(3) 1%	(5) 1.5%	0.73
Female	(290) 99%	(335) 98.5%	
Religion^b^			
Muslim	(71) 24.7%	(4) 1.2%	0.00
Christian	(215) 74.9%	(332) 98.8%	
Other	(1) .3%	(0) 0%	
Occupation^b^			
Self-employed	(91) 31.1%	(160) 47.3%	0.00
Civil Servant/Business	(28) 9.6%	(58) 17.2%	
Health worker	(7) 2.4%	(3) 0.9%	
Student	(28) 9.6%	(47) 13.9%	
Housewife/Farmer	(139) 47.4%	(70) 20.7%	
Location^b^			
Urban	(180) 61.4%	(244) 71.8%	0.01
Rural	(113) 38.6%	(96) 28.2%	
Region			
Northwest	(201) 68.6%	(241)70.9%	0.53
Southwest	(92) 31.4%	(99) 29.1%	
Child attended IWC elsewhere^b^			
Yes	(68) 23.2%	(36) 10.6%	0.00
No	(225) 76.8%	(304) 89.4%	
Mean Age of Caregiver			
	27.52	27.15	0.23
Mean Sum of Economic Variable^b^			
	1.68	1.92	0.00
Mean number of years of education			
	10.29	11.12	0.42
Number of months planning to breastfeed^b^			
	29.59	20.93	0.00

^a^Significant difference between caregivers with children 0–5 months at NIP and non-NIP sites, *p* < .05

^b^Significant difference between caregivers with children 6–8 months at NIP and non-NIP sites, *p* < .05

### Exclusive breastfeeding

Children 0–5 months at NIP sites were significantly more likely to be exclusively breastfed (90.2% vs. 56%, *p* = 0.00) (Table 2) compared to children at non-NIP sites. After adjusting for differences in religion, occupation, and number of months planning to breastfeed, children were almost seven times (Odds Ratio [OR]: 6.9; 95% Confidence Interval [CI]: 2.30, 21.09; β = 1.94) more likely to be exclusively breastfed at NIP sites compared to non-NIP sites (Table 3).

**Table 2:** Differences in Exclusive Breastfeeding, Wasting, Stunting, and Complementary Feeding in Children 0–5 and 6–8 Months

Children 0–5 months		
NIP = 61 (*n*) %	Non-NIP = 75 (*n*)%	(*X* ^*2*^ *) p*
Exclusive Breastfeeding		
(55) 90.2%	(42) 56%	(19.36) 0.00^a^
Wasting		
(5) 8.2%	(0) 0%	(4.87) 0.03^b^
Stunting		
(13) 21.3%	(26) 34.7%	–
Complementary Feeding		
–	–	–
Children 6–8 months		
NIP = 293 (*n*) %	Non-NIP = 340 (*n*) %	(*X* ^*2*^ *) p*
Exclusive Breastfeeding		
–	–	–
Wasting		
(8) 2.7%	(9) 2.6%	(0.00) 0.95
Stunting		
(37) 12.6%	(164) 48.2%	(92.08) 0.00^c^
Complementary Feeding		
(208) 71%	(232) 68.2%	(0.56) 0.45

^a^Significant for children 0–5 months at *p* < .00

^b^Significant for children 0–5 months at *p* < .05

^c^Significant for children 6–8 months at *p* < .00

**Table 3:** Logistic Regression Estimates and 95% CIs for Stunting, Wasting, and Exclusive Breastfeeding for Children 0–5 months

	B (CI)	SE	*p*	Exp (B)
Unadjusted				
Exclusive Breastfeeding^a^	1.94 [1.10, 3.25]^**^	0*.*98	0.00	6.94 [2.66, 18.12]
Wasting^b^	−18.82 [−19.64, −17.25]^**^	0.54	0.00	0*.*00 [0.00, 0.00]
Stunting^c^	0.73 [0.02, 1.60]	0.41	0.06	2.08 [0.94, 4.59]
Adjusted				
Exclusive Breastfeeding^a^	1.94 [1.04, 3.93]^**^	1.25	0.00	6.97 [2.30, 21.09]
Wasting^b^	*−*19.09 [−99.09, −16.75]	19.84	0.05	0.00 [0.00, 0.00]
Stunting^c^	0.73 [−0.21, 1.97]	0.55	0.15	2.07 [0.82, 5.23]

Adjusted for occupation, religion, number of months planning to breastfeed

*Abbreviations*: *CI* confidence interval, *SE* standard error

^**^ *p* < 0.001

^a^Base for exclusive breastfeeding is health worker, Christian, non-NIP

^b^Base for wasting is farmer/housewife, Christian, and NIP

^c^Base for stunting is health worker, Muslim, NIP

### Wasting

Children 0–5 months at NIP sites were significantly more likely to be wasted at NIP sites compared to non-NIP sites (8.2% vs. 0%, p=. 04) (Table 2). However, after adjusting for demographic differences, children at NIP sites did not have increased odds of being wasted (OR: .00; CI: .00, .00; β = −19.09) (Table 3). There were no significant differences in wasting in children 6–8 months between NIP and non-NIP sites (2.7, 2.6% respectively, *p* = .95) (Table 2). After adjusting for demographic differences children 6–8 months at non-NIP sites were 1.3 times more likely to be wasted, but this was not a significant difference (OR: 1.3; CI: 0.37, 4.65; β = 0.26) (Table 4).

**Table 4:** Logistic Regression Estimates and 95% CIs for Stunting, Wasting, and Complementary Feeding for Children 6–8 months

	B (CI)	SE	*p*	Exp (B)
Unadjusted				
Wasting^a^	−0.20 [−1.36, .98]	0.59	0.68	0.82 [0.30, 2.21]
Stunting^b^	1.89 [1.49, 2.38]^**^	0.23	0.00	6.62 [4.30, 10.18]
Complementary Feeding^c^	0.07 [−0.27, .42]	0.17	0.64	1.08 [0.75, 1.53]
Adjusted				
Wasting^a^	0.26 [−1.57, 2.56]	1.02	0.79	1.30 [0.37, 4.65]
Stunting^b^	1.71 [1.23, 2.32]^**^	0.28	0.00	5.52 [3.37, 9.02]
Complementary Feeding^c^	0.09 [−0.36, 0.57]	0.22	0.65	1.09 [0.72, 1.66]

Adjusted for occupation, religion, number of months planning to breastfeed, attended IWC elsewhere, Biological Mother or Father, urban location, economic status

*Abbreviations*: *CI* confidence interval, *SE* standard error

^**^ *p* < 0.001

^a^Base for wasting is student, Christian, Biological Mother, Rural location, NIP, attended IWC elsewhere

^b^Base for stunting is health worker, Christian, Biological Mother, Urban location, NIP, did not attend IWC elsewhere

^c^Base for complementary feeding is student, Christian, Biological Mother, Rural location, non-NIP, attended IWC elsewhere

### Stunting

Children 6–8 months were significantly more likely to be stunted at non-NIP compared to NIP sites (48.2% vs. 12.6%, *p* = .00) (Table 2). After adjusting for differences in occupation, religion, number of months planning to breastfeed, rural environment, attending other Infant Welfare Clinics, economic status, and non-biological caregiver, children were five times (OR: 5.5; CI: 3.37, 9.02; β = 1.71) more likely to be stunted at non-NIP sites compared to non-NIP sites (Table 4). There were no significant differences in stunting in children 0–5 months between NIP and non-NIP sites (21.3, 34.7% respectively, *p* = .09) (Table 2). After adjusting for demographic differences children 0–5 months at non-NIP sites were two times more likely to be stunted, but this was not significant (OR: 2.1; CI: 0.82, 5.23; β = 0.73) (Table 3).

### Complementary feeding

There were no significant differences in timely introduction of complementary foods between children at NIP and non-NIP sites (71% vs. 68.2%, *p* = .45) (Table 2). After adjusting for demographic differences children at NIP sites were more likely to be complementary fed, but not significantly different from children at non-NIP sites (OR: 1.1; CI: 0.72, 1.66; β = 0.09) (Table 4).

## Discussion

The results of this cross-sectional study suggest that caregivers who receive nutrition counseling services are more likely to practice exclusive breastfeeding and their children are less likely to be stunted. This is similar to a meta-analysis which found that as a result of nutrition counseling, caregivers were more likely to improve children’s daily energy intake, feeding frequency, and dietary intake [7] and a Peruvian study which found nutrition education provided by hospital workers reduced the rate of stunting and improved feeding practices [26].

The statistical difference of wasting in children 0–5 months was unexpected. Upon further analysis it was determined that all 5 cases of wasting came from one hospital in the SW Region of Cameroon. The hospital is one of very few health facilities with nutrition counseling services in the area and most cases of malnutrition from nearby health facilities are referred to that hospital. The hospital records show that although infants who first present are wasted, after further nutrition counseling they recover and no longer show signs of wasting.

The improved exclusive breastfeeding practices is in accordance with systematic reviews which found counseling or educational interventions increase exclusive breastfeeding by 43% at day 1, 30% at 1 month, 90% from 1 to 5 months [27, 28] and interventions offered through health services have the highest impact compared to interventions delivered only through the community or work environment [27]. Considering the manifold benefits for the mother and child from exclusive breastfeeding, it is very encouraging that 90% of the caregivers at NIP sites practiced exclusive breastfeeding, based on 24-h hour dietary intake, providing evidence that this form of breastfeeding promotion is effective in two regions of the country and should be scaled up to other regions.

There were a higher percentage of children 6–8 months at NIP sites who received timely introduction of complementary foods compared to children at non-NIP sites. However, the differences were small and not significant. In the future it would be useful to determine if there are also differences in dietary diversity at NIP and non-NIP sites considering iron-deficiency anemia is widespread throughout Cameroon [16] and NIP nutrition counselors educate caregivers on eating a diverse diet from locally-available foods.

Since this was a cross-sectional study it is not possible to determine the impact of nutrition counseling on nutritional status, particularly because nutritional status is influenced by a variety of factors. However, it is promising that there were significantly fewer children 6–8 months who were stunted at NIP sites. In this study, only 12.6% of children at NIP sites were stunted, which is far below the national 40% [15] and the non-NIP sites 48.2% prevalence. The significant differences in stunting between children at NIP and non-NIP remained, even after controlling for demographic differences, such as occupation, religion, economic status, and attending Infant Welfare Clinics at various health centers or hospitals. This difference could be attributed to the education caregivers at NIP sites receive at IWCs about complementary feeding practices, such as enriching the child’s cereal with protein foods and providing the child with his or her own plate and spoon. This additional nutrition education is valuable since breastfeeding promotion must be combined with education about timely introduction of appropriate complementary foods to prevent malnutrition [27].

For the future, the training for nutrition counselors at a clinic setting needs be combined with training peer counselors to provide breastfeeding support and education in the community, as research has shown that the highest increase in exclusive breastfeeding occurs when intervention occur concurrently in the community environment and through the health system [28]. One of the strengths of the Nutrition Improvement Program is the integration of nutrition counselors into ANC, maternity, PMTCT, and IWC, providing pregnant, recently delivered, and women with young children the same message in multiple settings. If NIP was expanded to the community level it is likely that infant and young child feeding practices would further improve.

Although the interest to address poor nutrition through international initiatives such as the SUN Movement and the second Sustainable Development Goal, “Zero Hunger”, are encouraging, such goals cannot be achieved without simultaneous investments in human and organizational capacity. Currently, most of the countries with the high burden of wasting and stunting are found in sub-Saharan Africa where there is a high dependency on external technical support to address nutrition-related problems [13]. CBCHS’ approach to training a cadre of nutrition counselors is one way to improve human capacity locally and decrease dependency on external technical support. Similar to other developing countries, there is a shortage of nurses and doctors in Cameroon, and since they are already overworked, it is unrealistic to expect them to provide appropriate and timely nutrition education and counseling. Developing a cadre of health workers working within hospitals and health centers and dedicated only to nutrition counseling and education is vital for contributing towards WHO’s priority action of increasing human resources to implement nutrition interventions.

It is important to note that although the differences in infant feeding and nutritional status described in this study are likely attributed to CBCHS’ investments in nutrition human and organizational capacity, in order for the results to be replicated and sustained in other parts of the country, there must also be investments in systemic capacity. Capacity development, as described by Potter and Bough (2004), covers a set of three separate, but interrelated elements such as individual capacity (the skills and tools), organizational capacity (the staff and structures) and the systemic capacity (systems, structures, and roles) [29]. Developing a workforce capacity is useful and paramount, but without similar investments in nutrition policies and systems, sustained, country-wide improvements in maternal and child nutrition will not be achieved.

### Limitations

This was a cross-sectional study; therefore, the results do not show causality. Since NIP evolved over time and started with very limited funding, no baseline information on IYCF or nutritional status was collected. In addition, health centers and hospitals in Cameroon do not regularly collect information on exclusive breastfeeding and complementary feeding; therefore, it is difficult to compare the results of this evaluation with pre-existing data. Furthermore, there were demographic differences between caregivers at NIP and non-NIP sites that may have influenced child feeding practices irrespective of nutritional counseling.

## Conclusion

Training and employing a cadre of health workers who are only tasked with nutrition counseling and integrating them into hospital services, such as the maternity, ANC, IWC, and PMTCT, is one way to improve human and organizational capacity and likely to effectively provide nutrition interventions promoting optimal child feeding in a low-resource setting. However, since this was a comparative cross-sectional evaluation, the positive results for increased exclusive breastfeeding and decreased stunting seen within NIP cannot be fully attributed to the nutrition program and future research is needed to confirm these results. Furthermore, more work needs to be done in Cameroon to also improve the systematic capacity to achieve goals set out in WHO’s Comprehensive Implementation Plan to Improve Maternal, Infant, and Young Child Nutrition.

## Additional file

Additional file 1: Infant Feeding Survey for Children 0-5 Months and 6-8 Months. (PDF 546 kb)
