# Erratum to: The potential effectiveness of the nutrition improvement program on infant and young child feeding and nutritional status in the Northwest and Southwest regions of Cameroon, Central Africa

**DOI:** 10.1186/s12913-017-2109-3

**Published:** 2017-02-24

**Authors:** Kate Reinsma, Godlove Nkuoh, Emmanuel Nshom

**Affiliations:** 1 Nutrition Improvement Program, Cameroon Baptist Convention Health Services, P.O Box 1, Nkwen, Bamenda, Cameroon; 2 AIDS Care and Prevention Program, Cameroon Baptist Convention Health Services, Bamenda, Cameroon

## Erratum

Following the publication of this article [1] it was brought to our attention that there is a recurring error in the ‘Results’ section of the ‘Abstract’ and the ‘Stunting’ section of the ‘Results’.

The sentence: “…children were five times (OR: 5.5; CI: 3.37, 9.02; β = 1.71) more likely to be stunted at non-NIP sites compared to non-NIP sites” should instead read: “… children were five times more likely to be stunted at non-NIP sites compared to NIP sites”.
