# Supplementary material for: The potential effectiveness of the nutrition improvement program on infant and young child feeding and nutritional status in the Northwest and Southwest regions of Cameroon, Central Africa
Source: BMC Health Serv Res. 2016 Nov 15;16:654. doi: 10.1186/s12913-016-1899-z (PMC5109805; doi:10.1186/s12913-016-1899-z)
Supplement: Additional file 1: — Infant Feeding Survey for Children 0-5 Months and 6-8 Months. (PDF 546 kb) [file 12913_2016_1899_MOESM1_ESM.pdf]

Section 0: Respondent Screening for Children 0-5 Months

Participant's Phone Number______________**Interviewer's Name**:________________ **Survey Site**____________**Survey Result** _____(1=complete; 2= partially complete; 3=refused to take the survey)
Date of Interview ____/____/___ **Identification Number:** __**Data Entry Clerk Name**:__________________
Date Entered: ____/____/___

INSTRUCTIONS TO INTERVIEWER: *IF THE WOMEN GAVE VERBAL CONSENT TO THE SURVEY, ASK THESE QUESTIONS IN A PRIVATE AREA. IF SHE DOES NOT WANT TO ANSWER SOME OF THE QUESTIONS, REASSURE HER THAT SHE CAN REFUSE TO ANSWER ANY OF THEM*

No.	QUESTIONS AND FILTERS	CODING CATEGORIES	SKIP TO
**Q001**	*Do you have a child who is less than 6 months old?*<br>**IF INFANT IS 6 MONTHS OR MORE, THE MOTHER SHOULD NOT BE INTERVIEWED WITH THIS QUESTIONNAIRE**	YES …………………………………..01<br>NO...00	
**Q002**	*What is the date of birth of the child?*<br>**IF THERE IS NO DOCUMENT SHOWING THE CHILD'S DOB, ASK THE MOTHER OR CAREGIVER IF SHE KNOWS THE CHILD'S DOB. RECORD HER RESPONSE.**<br><br>**IF YOU CANNOT OBTAIN DOB FROM A CARD OR THE MOTHER, YOU WILL NEED AND ASK THE MOTHER HOW OLD THE CHILD IS.**	____/____/_____<br>DD MM YY	
**Q003**	*Source for date of birth*<br>**CARD COULD BE AN IDENTIFICATION CARD, A HEALTH OR IMMUNIZATION CARD, A BIRTH CERTIFICATE OR A BAPTISMAL CERTIFICATE**	**Circle number not responses**<br>CARD ------------------------------------→ 1<br>CAREGIVER-----------------------------→ 2<br>DON'T KNOW---------------------------→ 8	
**Q004**	*How old are you?*<br>**IF CAREGIVER IS OLDER THAN 60 YEARS OR YOUNGER THAN 21 DO NOT CONTINUE. IF THE MOTHER IS OLDER THAN 60 YEARS OR YOUNGER THAN 18 YEARS DO NOT CONTINUE.**	[ ][ ]<br>AGE IN YEARS<br>DON'T KNOW……………….……....98<br>NO RESPONSE…………….........…...99	
**Q005**	*Have you been interviewed about breastfeeding and child feeding in the last five months?*	YES ……………………………….…01<br>NO...00	**→Q007**
**Q006**	*Where were you interviewed?*<br><br>**IF CAREGIVER NAMED ANY OF THE SITES LISTED IN THE COLUMN TO THE RIGHT THANK HIM OR HER AND DO NOT CONTINUE**	BANSO BAPTIST HOSPITAL…............01<br>NW REGIONAL HOSPITAL.……...........02<br>MBINGO BAPTIST HOSPITAL.............. 03<br>BELO BAPTIST HEALTH CENTER.......04<br>BANGOLAN HEALTH CENTER ....05<br>CDC MUKONJE HEALTH CENTER.......06<br>BAPTIST HOSPITAL MUTENGENE.......07<br>TIKO HOLFORT CMA..............................08	
**Q007**	*Has the child attended IWC elsewhere or been to any other health facilities?*	YES …………………………………..01<br>NO...00	

CLINIC-BASED SURVEY

FOR USE WITH

CAREGIVERS OF INFANTS AGE 0 TO LESS THAN 6 MONTHS OLD

Asking questions and recording answers

With the exception of questions 401-401, all questions in this module are based on mothers' (or other caregivers') recall. It is very important that you ask each question exactly as it is written on the questionnaire. If the mother or caregiver does not understand the question, you may need to use extra probing questions. Probing questions are discussed during interviewer training. It is important that all interviewers use the same probing questions.

In addition to the questions, there are statements that appear in all capital letters. These are interviewer instructions, and should not be read aloud to the mother/caregiver.

Most questions have pre-coded responses. It is important that you do not read these choices aloud. When you ask a question, you should listen to the mother's/caregiver's answer, then circle the code next to the category that best matches her answer.

Q.201: Ever breastfed child

In this question it does not matter how long the mother breastfed the child, only whether or not she ever gave the child the breast milk.

Q.203: Currently breastfeeding

For Question 203, it does not matter if the mother is giving (NAME) other liquids or foods as well as breast milk; what is of interest is if the infant or child is breastfeeding at all. (NAME refers to child's name.)

Q.204: Duration of breastfeeding

This question is only for mothers who are no longer breastfeeding the infant or child in question. It is important to try to get as accurate information as possible. If the caregiver says she cannot remember how long she breastfed the infant or child, urge her to think about it for a while, or ask her if she remembers how old the infant or child was when she completely stopped breastfeeding him/her. If the mother gives an approximate answer, such as "about one year," establish if it was exactly one year or how much more or less.

Record the number of months when the mother completely stopped breastfeeding the infant or child. It does not matter if she was giving the infant or child other liquids or foods in addition to breast milk; you are simply recording how many months she breastfed the infant or child.

Qs.211, 212 Other Liquids and foods given

The main purpose of questions 211 and 212 is to obtain a better picture of the diversity of the child's diet. You will ask the mother or other caregiver about the types of liquids and foods given to the child the day preceding the interview ("yesterday during the day or at night").

Question 211 asks about different liquids and foods given yesterday. It is important to ask about all the different kinds of liquids and foods. One reason is that for almost all infants less than 6 months old, only breast milk is recommended. We need to know if young infants are getting other liquids.

Read the question slowly and then read the liquids and foods in the list. Wait for the mother's/caregiver's response after each liquid or food and record whether the infant or child had each liquid (or group of liquids).

Question 212 asks about other liquids and foods the caregiver gives to the child, but may not have given yesterday. Do not read out these foods, but ask if the caregiver has ever given other foods or liquids besides breast milk.

Some foods in the list are listed as a single item – for example, vegetables – but may usually be eaten in a sauce, soup or stew. If the infant or child has eaten a mixed food like a sauce, soup, or stew, record all the food groups in the mixed food. For example, if the child ate a stew of beans, tomatoes, and green leaves, there should be a check mark for each of the three food groups that contain these foods.

Do not check off foods that have been added in very small amounts, or for seasoning. For example, if a spoon of pepe is added to a pot of stew, do not record that the infant or child has eaten vegetable. If one chili pepper is included in the family pot, do not record that as an "other fruit or vegetable."

ID #

	I WILL START WITH A FEW QUESTIONS ABOUT YOUR OWN BACKGROUND		
**NO.**	**QUESTONS AND FILTERS**	**CODING CATEGORIES**	**SKIP**
**Q101**	*Is the respondent male or female?*	FEMALE....01<br>MALE.... 02	
**Q102**	*Are you the child's biological mother or father?*	NO....00<br>YES....01	
**Q103**	*How many years of school have you completed, without repeating?*	[ ][ ]<br>YEARS COMPLETED<br>DON'T KNOW.... 98<br>NO RESPONSE....99	
**Q104**	*What is your religion?*	CHRISTIAN....01<br>MUSLIM....02<br>TRADITIONAL....03<br>NO RELIGION....04<br>OTHER (SPECIFY): ____________ 05	
**Q105**	*What is your main occupation?*<br><br>**WHAT KIND OF WORK DO YOU DO MOST OF THE TIME?**	SELF-EMPLOYED....01<br>CIVIL SERVANT....02<br>HEALTH WORKER....03<br>TECHNICIAN....04<br>FARMER....05<br>STUDENT.... 06<br>OTHER (SPECIFY): ____________07<br>DON'T KNOW ....98<br>NO RESPONSE 99	
**Q106**	*What is your marital status?*	MARRIED....01<br>COHABITING.... 02<br>SINGLE....03<br>WIDOWED....04<br>SEPARATED/DIVORCED....05<br>DON'T KNOW....98<br>NO RESPONSE....99	
**Q107**	*Which of the following items does your family own? (Circle all that apply)*	Y= YES, N=NO<br>Y N<br>RADIO....1 0<br>TELEVISION....1 0<br>MOTORBIKE....1 0<br>CAR....1 0	
**Q108**	*How many pregnancies have you had before?*<br><br>**ONLY ASK TO FEMALE PARTICIPANTS**	NONE....00<br>ONE....01<br>TWO....02<br>THREE....03<br>FOUR....04<br>MORE THAN FIVE....05<br>OTHER, SPECIFY....06<br>DON'T KNOW....98<br>NO RESPONSE 99	**→Q201**
**Q109**	*Out of the pregnancies how many children are alive?*	NONE....00<br>ONE....01<br>TWO....02<br>THREE....03<br>FOUR....04<br>FIVE....05<br>MORE THAN FIVE....06<br>OTHER, SPECIFY:____________07<br>DON'T KNOW....98<br>NO RESPONSE....99	

Section 2: Infant Feeding

No.	Questions and filters	Coding categories	Skip to
NOW I'D LIKE TO ASK YOU A FEW QUESTIONS ABOUT (NAME)			
		**Circle number not responses**	
**Q201**	*Has [NAME] ever breastfed?*	YES ……………………………..……01<br>NO…………….............………………00	**→203**
**Q202**	*What was the main reason [NAME] was never breastfed?*<br><br>**DO NOT READ RESPONSES**	MOTHER ILL/WEAK..01<br>CHILD ILL/WEAK…………………………........02<br>NIPPLE/BREAST PROBLEM…………………...03<br>INSUFFICIENT MILK ……………………..…04<br>MOTHER WORKING/PLANNING TO WORK...05<br>CHILD REFUSED ……………………….…...06<br>STARTED CONTRACEPTIVES………………...07<br>WORRIED ABOUT HIV/AIDS…………………08<br>DOCTOR'S ADVICE ……………………………09<br>OTHER HEALTH PROVIDER'S ADVICE….….10<br>SPECIFY:_______________<br>ADVICE OF FAMILY MEMBER/FRIENDS …...11<br>OTHER, SPECIFY: _______________12<br>DON'T KNOW…………………………………...98<br>NO RESPONSE …………………………………99	**→301**<br>**→301**<br>**→301**<br>**→301**<br>**→301**<br>**→301**<br>**→301**<br>**→301**<br>**→301**<br><br>**→301**<br>**→301**<br>**→301**<br>**→301**
**Q203**	*Is [NAME] still receiving breastfed milk?*	YES.……………………………....……………..01<br><br>NO……………...…………..………………………00	 <br>**→205**
**Q204**	*How long do you think [NAME] will take breast milk?*	NUMBER OF MONTHS ……………….[____|____]<br><br>DON'T KNOW ……..……………………………98	**→207**<br><br>**→207**
**Q205**	*For how many months was [NAME] breastfed?*<br><br>**IF LESS THAN 1 MONTH, RECORD '0'.**	NUMBER OF MONTHS ……………….[____|____]<br><br>DON'T REMEMBER ……………………………98	
**Q206**	*Why did you or the mother stop breastfeeding [NAME]?*<br><br>**IF MORE THAN ONE ANSWER GIVEN, PROBE FOR MAIN REASON.**<br><br>**DO NOT READ RESPONSES.**	MOTHER ILL/WEAK..01<br>CHILD ILL/WEAK…………………………........02<br>NIPPLE/BREAST PROBLEM…………………...03<br>INSUFFICIENT MILK ……………………..…04<br>MOTHER WORKING/PLANNING TO WORK...05<br>CHILD REFUSED ……………………….…...06<br>STARTED CONTRACEPTIVES………………...07<br>WORRIED ABOUT HIV/AIDS…………………08<br>DOCTOR'S ADVICE ……………………………09<br>OTHER HEALTH PROVIDER'S ADVICE….….10<br>SPECIFY:<br>ADVICE OF FAMILY MEMBER/FRIENDS …...11<br>OTHER, SPECIFY: _______________12<br>DON'T KNOW…………………………………...98<br>NO RESPONSE ……………………………......99	
**Q207**	*Did you or the mother receive assistance to start breastfeeding?*	YES……………………………………..……01<br>NO………………………………………………00	 **→210**

**Q208**	*By who?*<br><br>**CIRCLE YES FOR ANY PERSON WHO PROVIDED ASSISTANCE**	Y= YES, N=NO<br>Y N<br>NURSE/MIDWIFE……………........................…1 0<br>NUTRITION COUNSELOR…………...............1 0<br>DOCTOR..............……………………........….1 0<br>FAMILY MEMBER……………........……….....1 0<br>PEER EDUCATOR……………………………1 0<br>HUSBAND..........................………….....…….1 0<br>OTHER, SPECIFY_______________ ....……...1 0	
**Q209**	*What information did they provide?*<br>**CIRCLE YES FOR ANY TYPE OF INFORMATION PROVIDED**	Y= YES, N=NO<br>Y N<br>ATTACHMENT TO BREAST…....................…1 0<br>HOW TO STIMULATE BREASTFMILK…......1 0<br>WHAT TO EAT.OR DRINK...……...….......…..1 0<br>OTHER, SPECIFY_______________ ....……...1 0	
**Q210**	*Since yesterday has [NAME] received breast milk from the mother or any other person?*	YES ....01<br>NO...,...................................00<br>DON'T KNOW...98	
**Q211**	*Since yesterday has [NAME] received any of these things?*<br><br>**INTERVIEWER SHOULD ASK ABOUT EACH OPTION AND RECORD THE ANSWER. AFTER ASKING ALL OPTIONS, THEN ASK "DID THE BABY EAT ANYTHING ELSE DURING THE PAST 24 HOURS?"**	(Y=YES, N=NO)<br>Y N<br>A.VITAMIN DROPS/MEDICINE ....1 0<br>B. ORS...1 0<br>C. PLAIN WATER..1 0<br>D. SUGAR WATER/ SWEET DRINKS..........1 0<br>E. PALM WINE...1 0<br>F. FRUIT JUICE...1 0<br>G. TEA..1 0<br>H. PAP..1 0<br>I. INFANT FORMULA (GUIGOZ).................1 0<br>J. CERELAC..1 0<br>K. FRESH MILK FROM COWS.....................1 0<br>L. LIQUID OR POWDERED MILK................1 0<br>M. YOGURT...1 0<br>N. CUSTARD POWDER................................1 0<br>O.COOKED, MASHED FOODS. (FUFU).......1 0<br>P. RICE.. 1 0<br>Q. FRUITS... 1 0<br>R. VEGETABLES..1 0<br>S. BEANS ....1 0<br>T. EGG...1 0<br>U. STEW..1 0<br>V.OTHER LIQUIDS OR FOODS, SPECIFY..1 0<br>_______________________________	

**Q212**	*Is the baby taking anything else in addition to breast milk?*<br><br>**DO NOT READ OUT, PROBE AND CIRCLE A CODE FOR EACH**	(Y=YES, N=NO)<br>Y N<br>A.VITAMIN DROPS/MEDICINE ....1 0<br>B. ORS...1 0<br>C. PLAIN WATER..1 0<br>D. SUGAR WATER/ SWEET DRINKS...........1 0<br>E. PALM WINE..1 0<br>F. FRUIT JUICE..1 0<br>G. TEA...1 0<br>H. PAP...1 0<br>I. INFANT FORMULA (GUIGOZ)..................1 0<br>J. CERELAC...1 0<br>K. FRESH MILK FROM COWS......................1 0<br>L. LIQUID OR POWDERED MILK................1 0<br>M. YOGURT...1 0<br>N. CUSTARD POWDER.................................1 0<br>O.COOKED, MASHED FOODS. (FUFU).......1 0<br>P. RICE...1 0<br>Q. FRUITS..1 0<br>R. VEGETABLES...1 0<br>S. BEANS ....1 0<br>T. EGG..1 0<br>U. STEW...1 0<br>V.OTHER LIQUIDS OR FOODS, SPECIFY..1 0<br>________________	

Section 3: Client Satisfaction

**Q301**	*At today's visit or during your stay in the health unit, did anyone talk to you about how to feed the baby?*	YES...01<br>NO...00<br>DON'T KNOW...98	➨ **401**
**Q302**	*What type of health worker talked to you?*<br><br>**CIRCLE YES FOR ANY HEALTH WORKER WHO TALKED TO THE CAREGIVER**	Y= YES, N=NO<br>Y N<br>NURSE/MIDWIFE………………......................1 0<br>NUTRITION COUNSELOR…………...............1 0<br>DOCTOR..............………………………........…..1 0<br>PEER EDUCATOR……………………………1 0<br>**OTHER, SPECIFY_______________...……...1 0**	
**Q303**	*Were you satisfied with the information you received about how to feed [NAME]?*	VERY SATISFIED...03<br>SATISFIED...02<br>UNCERTAIN..01<br>NOT SATISFIED..00	

Section 4: Anthropometrics

If the child was weighed at today's clinic visit, ask to look at the child's clinic book and enter the child's weight and height below. If the child was not weighed and measured, weigh and measure the child.

Recumbent length will be measured to the nearest millimeter using horizontal boards. **All infant weight and length measurements should be taken in duplicate.** *If differences between measurements exist, a third measurement will be taken to ensure accuracy. Weights must agree within 0.1 kg.; length to the nearest .1 cm.*

**Q401**	*May I weigh (Name)?*	YES....01<br>NO....00<br><br>|___||___|. |___| KILOGRAMS	 ➨ **END**
**Q402**	*May I take (Name)'s length?*	YES....01<br>NO....00<br><br>|___||___||___| . |___| CM	 ➨ **END**

Section 5: General remarks from respondent

If the respondent has makes any comments or asks any questions please record them here in verbatim:

____
____
____
____
____
____

That is the end of our questionnaire. Thank you very much for taking time to answer these questions. We appreciate your help. Please do not discuss the details of this interview with anybody for the next two weeks. This is because we intend to interview other people about the same issues. If asked by a friend, neighbor or relative about what we discussed please tell them it was about infant feeding and nutrition counseling.

ID #

Section 0: Respondent Screening for Children 6-8 Months

Participant's Phone Number______________**Interviewer's Name**:________________ **Survey Site**____________**Survey Result** _____(1=complete; 2= partially complete; 3=refused to take the survey)
Date of Interview ____/____/___ **Identification Number:** __**Data Entry Clerk Name**:__________________
Date Entered: ____/____/___

INSTRUCTIONS TO INTERVIEWER: *IF THE WOMEN GAVE VERBAL CONSENT TO THE SURVEY, ASK THESE QUESTIONS IN A PRIVATE AREA. IF SHE DOES NOT WANT TO ANSWER SOME OF THE QUESTIONS, REASSURE HER THAT SHE CAN REFUSE TO ANSWER ANY OF THEM*

No.	QUESTIONS AND FILTERS	CODING CATEGORIES	SKIP TO
Q001	*Do you have a child who is at least 6 months old but less than 9 months old?*<br><br>**IF INFANT IS YOUNGER THAN 6 MONTHS OR IS 9 MONTHS 1 WEEK OLDER, THE MOTHER SHOULD NOT BE INTERVIEWED WITH THIS QUESTIONNAIRE**	YES …………………………………..01<br><br>NO...00	
Q002	*What is the date of birth of the child?*<br>**IF THERE IS NO DOCUMENT SHOWING THE CHILD'S DOB, ASK THE MOTHER OR CAREGIVER IF SHE KNOWS THE CHILD'S DOB. RECORD HER RESPONSE.**<br><br>**IF YOU CANNOT OBTAIN DOB FROM A CARD OR THE MOTHER, YOU WILL NEED AND ASK THE MOTHER HOW OLD THE CHILD IS.**	____/____/_____<br>DD MM YY	
Q003	*Source for date of birth*<br>**CARD COULD BE AN IDENTIFICATION CARD, A HEALTH OR IMMUNIZATION CARD, A BIRTH CERTIFICATE OR A BAPTISMAL CERTIFICATE.**	**Circle number not responses**<br>CARD ----------------------------------→ 1<br>CAREGIVER---------------------------→ 2<br>DON'T KNOW-----------------------→ 8	
Q004	*How old are you?*<br>**IF <u>CAREGIVER</u> IS OLDER THAN 60 YEARS OR YOUNGER THAN 21 DO NOT CONTINUE. IF THE <u>MOTHER</u> IS OLDER THAN 60 YEARS OR YOUNGER THAN 18 YEARS DO NOT CONTINUE.**	[ ][ ]<br>AGE IN YEARS<br>DON'T KNOW……………….……....98<br>NO RESPONSE…………….......…...99	
Q005	*Have you been interviewed about breastfeeding and child feeding in the last five months?*	YES ………………………………….01<br><br>NO...00	→Q007
Q006	*Where were you interviewed?*<br><br>**IF CAREGIVER NAMED ANY OF THE SITES LISTED IN THE COLUMN TO THE RIGHT THANK HIM OR HER AND DO NOT CONTINUE**	BANSO BAPTIST HOSPITAL…..........01<br>NW REGIONAL HOSPITAL……........02<br>MBINGO BAPTIST HOSPITAL.......... 03<br>BELO BAPTIST HEALTH CENTER....04<br>BANGOLAN HEALTH CENTER ....05<br>CDC MUKONJE HEALTH CENTER....06<br>BAPTIST HOSPITAL MUTENGENE....07<br>TIKO HOLFORT CMA..........................08	
Q007	*Has the child attended IWC elsewhere or been to any other health facilities?*	YES …………………………………..01<br><br>NO...00	

Infant Feeding Survey for Children 6-8 Months

CLINIC-BASED SURVEY
FOR USE WITH
CAREGIVERS OF INFANTS AGE 6 TO 8 MONTHS OLD
Asking questions and recording answers

With the exception of questions 401-401, all questions in this module are based on mothers' (or other caregivers') recall. It is very important that you ask each question exactly as it is written on the questionnaire. If the mother or caregiver does not understand the question, you may need to use extra probing questions. Probing questions are discussed during interviewer training. It is important that all interviewers use the same probing questions.

In addition to the questions, there are statements that appear in all capital letters. These are interviewer instructions, and should not be read aloud to the mother/caregiver.

Most questions have pre-coded responses. It is important that you do not read these choices aloud. When you ask a question, you should listen to the mother's/caregiver's answer, then circle the code next to the category that best matches her answer.

Q.201: Ever breastfed child

In this question it does not matter how long the mother breastfed the child, only whether or not she ever gave the child the breast.

Q.203: Currently breastfeeding

For Question 206, it does not matter if the mother is giving (NAME) other liquids or foods as well as breast milk; what is of interest is if the infant or child is breastfeeding at all. (NAME refers to child's name.)

Q.204: Duration of breastfeeding

This question is only for mothers who are no longer breastfeeding the infant or child in question. It is important to try to get as accurate information as possible. If the caregiver says she cannot remember how long she breastfed the infant or child, urge her to think about it for a while, or ask her if she remembers how old the infant or child was when she completely stopped breastfeeding him/her. If the mother gives an approximate answer, such as "about one year," establish if it was exactly one year or how much more or less.

Record the number of months when the mother completely stopped breastfeeding the infant or child. It does not matter if she was giving the infant or child other liquids or foods in addition to breast milk; you are simply recording how many months she breastfed the infant or child.

Qs.211, 214 Other Liquids and foods given

The main purpose of questions 211 and 214 is to obtain a better picture of the diversity of the child's diet. You will ask the mother or other caregiver about the types of liquids and foods given to the child the day preceding the interview ("yesterday during the day or at night").

Question 211 asks about different liquids and foods given yesterday. It is important to ask about all the different kinds of liquids and foods. One reason is that for almost all infants less than 6 months old, only breast milk is recommended. We need to know if young infants are getting other liquids.

Read the question slowly and then read the liquids and foods in the list. Wait for the mother's/caregiver's response after each liquid or food and record whether the infant or child had each liquid (or group of liquids).

Question 214 asks about other liquids and foods the caregiver gives to the child, but may not have given yesterday. Do not read out these foods, but ask if the caregiver has ever given other foods or liquids besides breast milk Some foods in the list are listed as a single item – for example, vegetables – but may usually be eaten in a sauce, soup or stew. If the infant or child has eaten a mixed food like a sauce, soup, or stew, record all the food groups in the mixed food. For example, if the child ate a stew of beans, tomatoes, and green leaves, there should be a check mark for each of the three food groups that contain these foods.

Do not check off foods that have been added in very small amounts, or for seasoning. For example, if a spoon of pepe is added to a pot of stew, do not record that the infant or child has eaten vegetable. If one chili pepper is included in the family pot, do not record that as an "other fruit or vegetable."

Qs. 212, 213 How many times infant or child ate yesterday

Question 212 asks at which age did the caregiver give the child solid/semi-solid foods. Ask the mother/caregiver the question just as it is written. You may need to use probes to help her remember at what age was food offered.

Solid/semi-solid foods include family foods, and also many special dishes prepared for infants. Thick soups and stews should be included. Thick paps and porridges are also included. Very thin, watery soups and gruels should not be included because infants and young children do not get enough energy (calories) from very thin soups and gruels.

Liquids do not count for this question. Also, very small snacks, such as a bite or two of someone else's food, should not be counted.

Question 213 asks about how many times the infant or child ate solid/semi-solid foods yesterday. Ask the mother/caregiver the question just as it is written. You may need to use probes to help her remember all the times her infant or child ate yesterday.

	**Section 1: Background Characteristics**		
	I WILL START WITH A FEW QUESTIONS ABOUT YOUR OWN BACKGROUND		
**NO.**	**QUESTONS AND FILTERS**	**CODING CATEGORIES**	**SKIP TO**
**Q101**	*Is the respondent male or female?*	FEMALE....01<br>MALE.... 02	
**Q102**	*Are you the child's biological mother or father?*	NO....00<br>YES....01	
**Q103**	*How many years of school have you completed, without repeating?*	[ ][ ]<br>YEARS COMPLETED<br>DON'T KNOW.... 98<br>NO RESPONSE....99	
**Q104**	*What is your religion?*	CHRISTIAN....01<br>MUSLIM....02<br>TRADITIONAL....03<br>NO RELIGION....04<br>OTHER (SPECIFY): ________ 05	
**Q105**	*What is your main occupation?*<br><br>**WHAT KIND OF WORK DO YOU DO MOST OF THE TIME?**	SELF-EMPLOYED....01<br>CIVIL SERVANT....02<br>HEALTH WORKER....03<br>TECHNICIAN....04<br>FARMER....05<br>STUDENT.... 06<br>OTHER (SPECIFY): ________07<br>DON'T KNOW ....98<br>NO RESPONSE 99	
**Q106**	*What is your marital status?*	MARRIED....01<br>COHABITING.... 02<br>SINGLE....03<br>WIDOWED....04<br>SEPARATED/DIVORCED....05<br>DON'T KNOW....98<br>NO RESPONSE....99	
**Q107**	*Which of the following items does your family own? (Circle all that apply)*	Y= YES, N=NO<br>Y N<br>RADIO....1 0<br>TELEVISION....1 0<br>MOTORBIKE....1 0<br>CAR....1 0	
**Q108**	*How many pregnancies have you had before?*<br><br>**ONLY ASK TO FEMALE PARTICIPANTS**	NONE....00<br>ONE....01<br>TWO....02<br>THREE....03<br>FOUR....04<br>MORE THAN FIVE....05<br>OTHER, SPECIFY:________06<br>DON'T KNOW....98<br>NO RESPONSE 99	**→Q201**
**Q109**	*Out of the pregnancies how many children are alive?*	NONE....00<br>ONE....01<br>TWO....02<br>THREE....03<br>FOUR....04<br>MORE THAN FIVE....05<br>OTHER, SPECIFY:________06<br>DON'T KNOW....98<br>NO RESPONSE 99	

Section 2: Infant Feeding

No.	Questions and filters	Coding categories	Skip to
NOW I'D LIKE TO ASK YOU A FEW QUESTIONS ABOUT (NAME)			
**Q201**	*Has [NAME] ever breastfed?*	YES ……………………………………...…….01<br>NO…………………...…………………………00	**→203**
**Q202**	*What was the main reason you or the mother did not ever breastfeed (NAME)?*<br><br>**DO NOT READ RESPONSES**	MOTHER ILL/WEAK..01<br>CHILD ILL/WEAK………………………….........02<br>NIPPLE/BREAST PROBLEM…………………...03<br>INSUFFICIENT MILK ……………………..…04<br>MOTHER WORKING/PLANNING TO WORK...05<br>CHILD REFUSED ……………………..…...06<br>STARTED CONTRACEPTIVES………………...07<br>WORRIED ABOUT HIV/AIDS………………….08<br>DOCTOR'S ADVICE ……………………………09<br>OTHER HEALTH PROVIDER'S ADVICE…..…10<br>SPECIFY:______________________________<br>ADVICE OF FAMILY MEMBER/FRIENDS …...11<br>OTHER, SPECIFY: ____________________12<br>DON'T KNOW………………………………...98<br>NO RESPONSE …………………………………99	**→301**<br>**→301**<br>**→301**<br>**→301**<br>**→301**<br>**→301**<br>**→301**<br>**→301**<br>**→301**<br><br><br>**→301**<br>**→301**<br>**→301**<br>**→301**
**Q203**	*Is [NAME] still receiving breast milk*	YES ……………………………...……………..……01<br><br>NO……………...…………….…...…………..…00	 <br>**→205**
**Q204**	*How long do you think [NAME] will take breast milk?*	NUMBER OF MONTHS ……………….[____|____]<br><br>DON'T KNOW ……..……………………………98	**→207**<br><br>**→207**
**Q205**	*For how many months was [NAME] breastfed?*<br><br>**IF LESS THAN 1 MONTH, RECORD '0'.**	NUMBER OF MONTHS ……………….[____|____]<br><br>DON'T REMEMBER ……………………………98	

**Q206**	*Why did you or the mother stop breastfeeding (NAME)?*<br><br>**IF MORE THAN ONE ANSWER GIVEN, PROBE FOR MAIN REASON.**<br><br>**DO NOT READ RESPONSES.**	MOTHER ILL/WEAK...01<br>CHILD ILL/WEAK…………………………........02<br>NIPPLE/BREAST PROBLEM…………………...03<br>INSUFFICIENT MILK ……………………..…04<br>MOTHER WORKING/PLANNING TO WORK...05<br>CHILD REFUSED ……………………….…...06<br>STARTED CONTRACEPTIVES………………...07<br>WORRIED ABOUT HIV/AIDS…………………08<br>DOCTOR'S ADVICE ……………………………09<br>OTHER HEALTH PROVIDER'S ADVICE…..…10<br>SPECIFY:______________________<br>ADVICE OF FAMILY MEMBER/FRIENDS …...11<br>OTHER, SPECIFY: ________________12<br>DON'T KNOW…………………………………...98<br>NO RESPONSE ………………………………....99	
**Q207**	*Did you or the mother receive assistance to start breastfeeding?*	YES……………………………………..……01<br>NO……………………………………………00	**→210**
**Q208**	*By who?*<br><br>**CIRCLE YES FOR ANY PERSON WHO PROVIDED ASSISTANCE**	Y= YES, N=NO<br>Y N<br>NURSE/MIDWIFE…………….....................…1 0<br>NUTRITION COUNSELOR…………..............1 0<br>DOCTOR..............……………………........….1 0<br>FAMILY MEMBER……………........……….....1 0<br>PEER EDUCATOR……………………………1 0<br>HUSBAND.........................……………..……...1 0<br>OTHER, SPECIFY_______________...……...1 0	
**Q209**	*What information did they provide?*<br><br>**CIRCLE YES FOR ANY TYPE OF INFORMATION PROVIDED**	Y= YES, N=NO<br>Y N<br>ATTACHMENT TO BREAST…....................…1 0<br>HOW TO STIMULATE BREASTFMILK….....1 0<br>WHAT TO EAT.OR DRINK...……..…......…..1 0<br>OTHER, SPECIFY_______________...……...1 0	
**Q210**	*Since yesterday has [NAME] received breast milk from the mother or any other person?*	YES ....01<br>NO...00<br>DON'T KNOW...98	

**Q211**	*Since yesterday has [NAME] received any of these things?*<br><br>**INTERVIEWER SHOULD ASK ABOUT EACH OPTION AND RECORD THE ANSWER. AFTER ASKING ALL OPTIONS, THEN ASK "DID THE BABY EAT ANYTHING ELSE DURING THE PAST 24 HOURS?"**	(Y=YES, N=NO)<br>Y N<br>A.VITAMIN DROPS/MEDICINE ....1 0<br>B. ORS..1 0<br>C. PLAIN WATER..1 0<br>D. SUGAR WATER/ SWEET DRINKS..........1 0<br>E. PALM WINE...1 0<br>F. FRUIT JUICE...1 0<br>G. TEA...1 0<br>H. PAP...1 0<br>I. INFANT FORMULA (GUIGOZ).................1 0<br>J. CERELAC...1 0<br>K. FRESH MILK FROM COWS......................1 0<br>L. LIQUID OR POWDERED MILK................1 0<br>M. YOGURT...1 0<br>N. CUSTARD POWDER...............................1 0<br>O.COOKED, MASHED FOODS. (FUFU).......1 0<br>P. RICE..1 0<br>Q. FRUITS..1 0<br>R. VEGETABLES..1 0<br>S. BEANS ....1 0<br>T. EGG...1 0<br>U. STEW..1 0<br>V.OTHER LIQUIDS OR FOODS, SPECIFY..1 0<br>________________________	
**Q212**	*At what age did you give baby other food in addition to breast milk?*<br><br>**SOLID, SEMI-SOLID, AND SOFT FOODS ARE DEFINED AS MUSHY OR SOLID FOODS, NOT FLUIDS**.	BABY IS NOT TAKING ANY SOLID FOODS.. 00<br><br>AGE IN MONTHS WHEN BABY FIRST TOOK. SOLID OR SEMI-SOLID FOODS.... [____|____]<br><br>DON'T KNOW..98	**→301**
**Q213**	*How many times did [NAME] eat foods other than liquids yesterday during the day or at night?*<br><br>**SMALL SNACKS AND SMALL FEEDS SUCH AS ONE OR TWO BITES OF MOTHER'S OR SIBLING'S FOOD SHOULD NOT BE COUNTED.**	CHILD NEVER TOOK SOLID, OR SEMI-SOLID FOOD..00<br>1 TIME...01<br>2 TIMES...02<br>3 TIMES...03<br>FOUR OR MORE TIMES...................................04<br><br>DON'T' KNOW...98	

**Q214**	*Is the baby taking anything else in addition to breast milk?*<br><br>**DO NOT READ OUT, PROBE AND CIRCLE A CODE FOR EACH**	(Y=YES, N=NO)<br>Y N<br>A.VITAMIN DROPS/MEDICINE ....1 0<br>B. ORS..1 0<br>C. PLAIN WATER..1 0<br>D. SUGAR WATER/ SWEET DRINKS..........1 0<br>E. PALM WINE...1 0<br>F. FRUIT JUICE..1 0<br>G. TEA..1 0<br>H. PAP..1 0<br>I. INFANT FORMULA (GUIGOZ).................1 0<br>J. CERELAC...1 0<br>K. FRESH MILK FROM COWS......................1 0<br>L. LIQUID OR POWDERED MILK................1 0<br>M. YOGURT...1 0<br>N. CUSTARD POWDER...............................1 0<br>O.COOKED, MASHED FOODS. (FUFU).......1 0<br>P. RICE...1 0<br>Q. FRUITS..1 0<br>R. VEGETABLES..1 0<br>S. BEANS ....1 0<br>T. EGG...1 0<br>U. STEW..1 0<br>V.OTHER LIQUIDS OR FOODS, SPECIFY..1 0<br>_______________	

Section 3: Client Satisfaction

**Q301**	*At today's visit or during your stay at the health center did anyone talk to you about how to feed the baby?*	YES..01<br>NO...00<br>DON'T KNOW...98	➨ **401**
**Q302**	*What type of health worker talked to you?*<br>**CIRCLE YES FOR ANY HEALTH WORKER WHO TALKED TO THE CAREGIVER**	Y= YES, N=NO<br>Y N<br>NURSE/MIDWIFE……………......................…1 0<br>NUTRITION COUNSELOR…………...............1 0<br>DOCTOR...............……………………........…..1 0<br>PEER EDUCATOR……………………………1 0<br>**OTHER, SPECIFY_______________....……...1 0**	
**Q303**	*Were you satisfied with the information you received about how to feed [NAME]?*	VERY SATISFIED..03<br>SATISFIED...02<br>UNCERTAIN...01<br>NOT SATISFIED..00	

Section 4: Anthropometrics

If the child was weighed at today's clinic visit, ask to look at the child's clinic book and enter the child's weight and height below. If the child was not weighed and measured, weigh and measure the child.

Recumbent length will be measured to the nearest millimeter using horizontal boards. ***All infant weight and length measurements should be taken in duplicate.*** *If differences between measurements exist, a third measurement will be taken to ensure accuracy. Weights must agree within 0.1 kg.; length t to the nearest .1 cm.*

**Q401**	*May I weigh (Name)?*	YES....................01<br>NO....................00 |___||___|. |___| KILOGRAMS	➨ END
**Q402**	*May I take (Name)'s height?*	YES....................01<br>NO....................00 |___||___||___| . |___| CM	➨ END

Section 5: General remarks from respondent

If the respondent has makes any comments or asks any questions please record them here in verbatim:

____

____

____

____

____

____

____

____

____

____

That is the end of our questionnaire. Thank you very much for taking time to answer these questions. We appreciate your help.
Please do not discuss the details of this interview with anybody for the next two weeks. This is because we intend to interview other people about the same issues. If asked by a friend, neighbor or relative about what we discussed please tell them it was about infant feeding and nutrition counseling.
